# A novel mutation of *ramR* involved in tigecycline resistance in *Klebsiella pneumoniae*

**DOI:** 10.1128/spectrum.03204-24

**Published:** 2025-05-15

**Authors:** Yuyao Xu, Qi Chen, Chenhao Zhao, Xin Ma, Lei Wang, Feinan Qian, Xiangyu Guan, Hong Du, Haifang Zhang

**Affiliations:** 1 Department of Clinical Laboratory, The Second Affiliated Hospital of Soochow University, Suzhou, Jiangsu, China; 2 Department of Laboratory Medicine, The Affiliated Zhangjiagang TCM Hospital of Yangzhou University, Zhangjiagang, Jiangsu, China; 3 MOE Key Laboratory of Geriatric Diseases and Immunology, Soochow University 12582, Suzhou, Jiangsu, China; Jinan University, Shenzhen, China

**Keywords:** tigecycline, *ramR*, *Klebsiella pneumoniae*, resistance

## Abstract

This study aims to investigate the novel potential tigecycline resistance mechanism in *Klebsiella pneumoniae* and provide new insights for the clinical treatment of infections caused by carbapenem-resistant *Klebsiella pneumoniae. In vitro* experiments were conducted to induce tigecycline resistance in *Klebsiella pneumoniae*, simulating potential genetic mutations that may arise under the selective pressure of tigecycline in clinical settings. Next-generation sequencing and real-time quantitative PCR (RT-qPCR) were employed to analyze bacterial genomic mutations and the transcription of related genes, respectively. Gene knockout technology, broth microdilution method, and resistance stability tests were utilized to further validate the function of the identified gene mutation sites. A novel two-base pair deletion mutation (position 517-518) in the *ramR* gene (g. 517_518 del) was identified in *Klebsiella pneumoniae* strains cultured in broth medium containing progressively increasing concentrations of tigecycline (4, 8, and 16 mg/L). The impact of this mutation on tigecycline resistance was characterized. The RT-qPCR analysis revealed a consistent upregulation of *ramR* expression across all tigecycline-induced strains, with significant elevation observed at various concentrations (0.5, 1, 2, 4, 8, and 16 mg/L). Furthermore, the g. 517_518 del mutation in *ramR* was associated with sustained tigecycline resistance. This study illustrated a novel *ramR* mutation, leading to tigecycline resistance in *Klebsiella pneumoniae*.

**IMPORTANCE:**

In this study, a novel missense mutation (g. 517_518 del GC) was detected in the *ramR* of tigecycline-induced *Klebsiella pneumoniae*, which was conducted *in vitro*, and the effects of anti-tigecycline caused by this mutation in *ramR* were confirmed. A high expression of *ramR* was observed in all tigecycline-induced strains. In addition, g. 517_518 del GC in *ramR* maintained tigecycline resistance. In summary, we illustrated a novel mutation of *ramR*, leading to tigecycline resistance in *Klebsiella pneumoniae*.

## INTRODUCTION

*Klebsiella pneumoniae*, a member of the *Enterobacterales*, is an opportunistic pathogen known for its strong environmental adaptability (1) and its ability to cause severe bloodstream infections (BSIs) (2), which result in increased morbidity and mortality (3). Recent studies have reported the emergence and widespread dissemination of multidrug-resistant hypervirulent *Klebsiella pneumoniae* (hvKP) clinical isolates (3). The clinical detection rate of carbapenem-resistant hvKP (CR-hvKP) is rising year by year (4), presenting new difficulties and challenges for clinical treatment.

Tigecycline is the first glycylcycline antibiotic approved by the US Food and Drug Administration (FDA) for clinical application (5) and remains one of the primary options for the effective treatment of severe carbapenem-resistant *Klebsiella pneumoniae* (CRKP) infections (6). In recent decades, tigecycline-resistant CRKP isolates have emerged worldwide (7), raising significant concerns. The simultaneous occurrence of hvKP infection with both carbapenem and tigecycline resistance can lead to more difficult clinical treatment, fewer available antibiotics, and a worse prognosis (8).

Tigecycline is the substrate of the AcrAB-TolC efflux pump (9). The expression of this pump is regulated by several proteins, including RamA, SoxS, RobA, and AcrR (10–12). Notably, the expression of RamA is regulated by the repression of RamR encoded by gene *ramR* (13).

In this study, *Klebsiella pneumoniae* ATCC 43816 was gradually induced under selective laboratory pressure from low to high concentrations of tigecycline to simulate the possible gene mutations that might occur under tigecycline pressure in clinical practice. Then, the potential mechanism of tigecycline resistance in *Klebsiella pneumoniae* was preliminarily explored to face the possible clinical emergence of drug-resistant strains. Identifying mutation information holds great significance for the treatment of *Klebsiella pneumoniae* infection.

## MATERIALS AND METHODS

### Bacterial strains, plasmids, and primers

As shown in Table 1, all strains were stored in 30% glycerol Luria-Bertani broth (LB) at −80°C in an ultra-low temperature freezer, with resuscitation conducted before use. The plasmids and primers used in this study are presented in Tables 2 and 3, respectively.

**TABLE 1:** Strains used in this study

Strains	Description	Source
*E. coli* DH5α	Cloning host	Laboratory stock
*K. pneumoniae* ATCC 43816	Wild type	Laboratory stock
DH5α-pSGKP	Rifampicin resistance	Laboratory stock
*E. coli* ATCC 25922	Quality control strain	Laboratory stock
ATCC 43816Δ*ramR*	ATCC 43816 *ramR*-deletion mutant	This study
ATCC 43816-pCas	ATCC 43816 with the empty vector pCas, apramycin resistance	This study
ATCC 43816-pBAD24	ATCC 43816 with the empty vector pBAD24, ampicillin resistance	This study
ATCC 43816Δ*ramR*-pBAD24	ATCC 43816Δ*ramR* with the empty vector pBAD24, ampicillin resistance	This study
ATCC 43816Δ*ramR*-pC*ramR*	ATCC 43816Δ*ramR* with the complement plasmid pC*ramR*, ampicillin resistance	This study
ATCC 43816Δ*ramR*-pCt*ramR*	ATCC 43816Δ*ramR* with the *ramR* mutant sequence plasmid, ampicillin resistance	This study

**TABLE 2:** Plasmids used in this study

Plasmids	Description	Source/reference
pCasKP	Temperature sensitive, apramycin resistance	(14)
pSGKP	Rifampicin resistance	(14)
pSGKP-RamR-N20	pSGKP with target sgRNA *ramR*-N20 (20 bp), rifampicin resistance	This study
pBAD24	L-arabinose-inducible expression plasmid, ampicillin resistance	Laboratory stock
pC*ramR*	*ramR* gene (657 bp) cloned in the expression vector pBAD24, ampicillin resistance	This study
pCt*ramR*	*ramR* mutant sequence (655 bp) cloned in the expression vector pBAD24, ampicillin resistance	This study

**TABLE 3:** Primers used in this study

Primers	Nucleotide sequence (5′→3′)	Function
Apr-F	TCGGTCAGCTTCTCAACCTT	Identify the transformants of pCasKP (ATCC 43816)
Apr-R	ACCAACTTGCCATCCTGAAG	
*Spe*I-RamR-gS	GGTACTAGTGTTACTGGAAGCTGCCACCGGTTTTAGAGCTAGAAATAGCAAGTT	Construct pSGKP with targeted sgRNA (pSGKP-RamR-N20)
*Xba*I-gRNA-R	GCCGCTCTAGAAGTAGTGGA	
RamR-US	CGCTTCCACCTGGCTAA	Construct a homologous recombination arm as the *ramR*-deletion repair template
RamR-UA	GGCGTCCAAACCGCCGATCTTGG	
RamR-DS	GCGGTTTGGACGCCAAATGACCC	
RamR-DA	CACCTGATGCCACGAAT	
RamR-W-S	TCATCAGGAAATCCCACC	Identify the correct Δ*ramR* mutants
RamR-W-A	CCTCACAGTTTACAGCACCT	
RamR-J-S	TCATCAGGAAATCCCACC	
RamR-J-A	CGACCTTAAACACGTCGTAC	
RamR-H-S	CCGGAATTCCAAGATCGGCGGTTTG	Construct and identify the recombinant plasmid pC*ramR* or pCt*ramR*
RamR-H-A	GCTCTAGAGGTGAGCGCAGGGAT	
RamR-N-S	TAGTGGCTCGTCCAAAGA	
RamR-N-A	CCAGCGACAGAAACAGG	
pSGKP	AGGATTTGCAGACTACG	Confirm pSGKP elimination
M13R	CAGGAAACAGCTATGACC	
q16s-FOR	CTACAAGACTCTAGCCTGCCAGTTTC	Primers for real-time fluorescence quantitative PCR amplification
q16s-REV	GCGGTCTGTCAAGTCGGATGTG	
RpoE-FOR	GCGAGCAGTTAACGGATCAGGTC	
RpoE-REV	CGGCGGTACATAGCGGGAAAC	
RamR-FOR	CGCCGCCAGGTAAAAGAGAGC	
RamR-REV	TTCCGCCAGCGACAGAAACAG	
RamR-TS	AGTGGCTCGTCCAAAGA	Confirm the mutation sites
RamR-TA	TCGGTAAACGGGTAGGT	
pBAD24-For	ACATTGATTATTTGCACGGCGT	Identify the plasmid pBAD24
pBAD24-Rev	CAGACCGCTTCTGCGTTCTG	

### Induction of resistance in tigecycline-sensitive *Klebsiella pneumoniae*

*Klebsiella pneumoniae* ATCC 43816, a tigecycline-sensitive strain, was cultured on LB agar medium and incubated at 37°C overnight. A single colony was inoculated into 3 mL of LB liquid medium and incubated at 37°C with shaking at 180 rpm for 12 h. Subsequently, 100 µL of the bacterial culture was transferred into 10 mL of fresh LB liquid medium and incubated under the same conditions until the logarithmic growth phase (OD_600_ = 0.8–1.0) was reached.

The minimum inhibitory concentration (MIC) of ATCC 43816 to tigecycline was determined to be 1 mg/L, and a subinhibitory concentration (0.5 mg/L) of tigecycline was chosen to start the induction.

For the induction process, 100 µL of logarithmic-phase bacterial culture (OD_600_ = 0.8–1.0) was evenly spread onto cation-adjusted Mueller-Hinton broth (CAMHB) solid medium supplemented with tigecycline at an initial concentration of 0.5 mg/L. The tigecycline concentration was doubled for each induction. The plates were inverted and incubated at 37°C for 24 h. After incubation, single colonies were selected and inoculated into 3 mL of CAMHB liquid medium containing the corresponding tigecycline concentration, followed by incubation at 37°C with shaking at 180 rpm for 24 h. This procedure was repeated every 24 h for 3 d at each concentration, and the MIC was measured to confirm the induction of resistance. On the final day of each concentration, single colonies were proportionally enriched until visible turbidity was observed. The omega E.Z.N.A. Bacterial DNA Kit was used for genome extraction. The induction process was terminated if bacterial growth exhibited flocculent characteristics.

### Drug susceptibility test

Test strains and the quality control strain *Escherichia coli* ATCC 25922 were inoculated into LB agar medium for overnight cultivation. Growth control wells exhibited at least 2 mm of bottom sediment or obvious turbidity, while negative control wells showed no bacterial growth and remained clear. The MIC values of the quality control strain ATCC 25922 to tigecycline ranged from 0.03 to 0.25 mg/L. The MIC was defined as the lowest concentration visibly inhibiting bacterial growth in each well. All experimental data were derived from at least three independent experiments to ensure stability and reliability.

The MIC for tigecycline was determined by using the broth microdilution method as outlined in the Clinical and Laboratory Standards Institute (CLSI) M07 standard (15). Due to the absence of CLSI breakpoints, tigecycline susceptibility was evaluated using FDA breakpoints. The FDA defines tigecycline MIC breakpoints for *Enterobacterales* as follows: susceptible ≤ 2 mg/L and resistant ≥ 8 mg/L (16).

### Next-generation sequencing and genome assembly

Whole genome sequencing of both the wild-type and induced strains was performed using the Illumina NovaSeq 6000 next-generation sequencing platform. The NEBNext Ultra DNA Library Prep Kit was employed to generate sequencing libraries. Index codes were added for Illumina sequencing, followed by PCR amplification. PCR products were purified using the AMPure XP system (Beverly, USA), evaluated for library quality on the Agilent 5400 system (Agilent, USA), and quantified by quantitative PCR (qPCR). Samples with concentrations exceeding 1.5 nmol/L were deemed suitable for subsequent sequencing on the designated platform. The sequencing data were then paired and assembled using SPAdes v.3.13.0 (17).

### Gene mutation analysis

Using the genome of the wild-type strain *Klebsiella pneumoniae* ATCC 43816 as a reference, Snippy v.4.6 was employed to identify base substitution, insertion, and deletion. The BLAST database was utilized to compare mutations at the protein level. RamR-TS and RamR-TA primer pairs amplified the mutant sequence, which was verified through Sanger sequencing.

### Homology modeling and mutation site labeling

The RamR amino acid sequence of *Klebsiella pneumoniae* ATCC 43816 was submitted to the SWISS-MODEL workspace for homology modeling, with subsequent editing performed using PyMOL 3D graphics software.

### Gene knockout

Tigecycline-sensitive *Klebsiella pneumoniae* ATCC 43816 was selected as the wild-type strain, and the CRISPR-Cas9 technology (18, 19) was used to knock out gene *ramR*. First, the clone *Klebsiella pneumoniae* ATCC 43816-pCas, harboring the plasmid pCasKP, was successfully isolated. Subsequently, the plasmid pSGKP-RamR-N20 was constructed to facilitate targeted gene editing. A homologous recombination arm was generated using overlap PCR. Following this, both pSGKP-RamR-N20 and the homologous recombination arm were introduced into the electrocompetent ATCC 43816-pCas cells via electroporation. Finally, the *ramR* gene deletion strain was confirmed through genetic validation, and the plasmids pSGKP and pCasKP were sequentially eliminated to ensure a clean genetic background. The TIANGEN TIANprep Mini Plasmid Kit was used for plasmid extraction, while the Axygen DNA Gel Extraction Kit facilitated gel extraction. DNA purification was performed with the TIANGEN TIANquick Midi Purification Kit. All these kits were supplied by TIANGEN Co. in China.

### Construction of complementary strains

Complementation plasmids containing the wild-type or mutant *ramR* gene were transferred into Δ*ramR*, with empty vectors serving as negative controls to assess the role of *ramR* gene mutation in tigecycline resistance in *Klebsiella pneumoniae*. Complementation plasmids pC*ramR* and pCt*ramR* were constructed and electroporated into the electrocompetent cells to generate complementation strains. Plasmid extraction, gel extraction, and DNA purification were performed following the same protocols as above.

### Total RNA extraction

Total bacterial RNA was extracted using the Vazyme FastPure Cell/Tissue Total RNA Isolation Kit V2. The concentration and purity of the extracted RNA were assessed, and RNA integrity was verified via electrophoresis, ensuring no residue remained in the wells. The experiment was repeated three times.

### Real-time quantitative PCR (RT-qPCR)

The expression level of *ramR* was analyzed using RT-qPCR. RNA reverse transcription was performed with the Vazyme HiScript III RT SuperMix kit, and the fluorescence quantitative reaction system was prepared after a fivefold dilution of reverse transcription products. The Vazyme Taq Pro Universal SYBR qPCR Master Mix kit was utilized. According to the principle of relative quantitative analysis, the 16S rRNA gene was regarded as the reference, with the *ramR* expression level of ATCC 43816 as the control. The relative expression of *ramR* was normalized to that of the 16S rRNA gene using the 2^-ΔΔCT^ method. For each specimen, three replicate wells were set up. The final results were based on the three independent experiments.

### Resistance stability testing

Pressure-selected resistant strains were inoculated on LB agar medium and incubated at 37°C overnight (16–18 h), termed primary bacteria. On the next day, single colonies were inoculated on new LB plates and incubated overnight at 37℃ for 24 h. The process was repeated 10 times, with drug-resistant strains passed continuously for 10 d (10 generations/12 h). Single colonies from different generations were selected for tigecycline MIC determination, and a line chart depicting the relationship between passage number and tigecycline MIC of strains was constructed to assess resistance stability.

### Statistical analysis

Ordinary one-way analysis of variance (ANOVA) was used to test for equality of means, and multiple comparisons were performed using Tukey’s test. Statistical analyses were executed using GraphPad Prism 9.0. *P* values of <0.05 were considered statistically significant.

## RESULTS

### Antimicrobial susceptibility testing of induced resistance strains

Prior to induction, the ATCC 43816 strain demonstrated susceptibility to tigecycline, with a baseline MIC of 1 mg/L, indicating its initial sensitivity to the antibiotic. Following induction, the MIC values of tigecycline in all strains (0.5, 1, 2, 4, 8, and 16 mg/L) increased to a range of 8–32 mg/L, demonstrating the development of tigecycline resistance (Table 4).

**TABLE 4:** MICs of *Klebsiella pneumoniae* to tigecycline

Isolate*^a^*	Inducer concentration (mg/L)	MIC (mg/L) (susceptibility)^*b*^
ATCC 43816	0	1 (S)
ATCC 43816-0.5	0.5	8 (R)
ATCC 43816-1	1	16 (R)
ATCC 43816-2	2	32 (R)
ATCC 43816-4	4	32 (R)
ATCC 43816-8	8	32 (R)
ATCC 43816-16	16	32 (R)

^*a*^ The number after "ATCC 43816-" indicates that the strain was induced with tigecycline of corresponding concentration.

^*b*^ S, susceptible; R, resistant.

### Sequence alignment analysis of *ramR* in tigecycline-resistant strains

**Fig 1:**
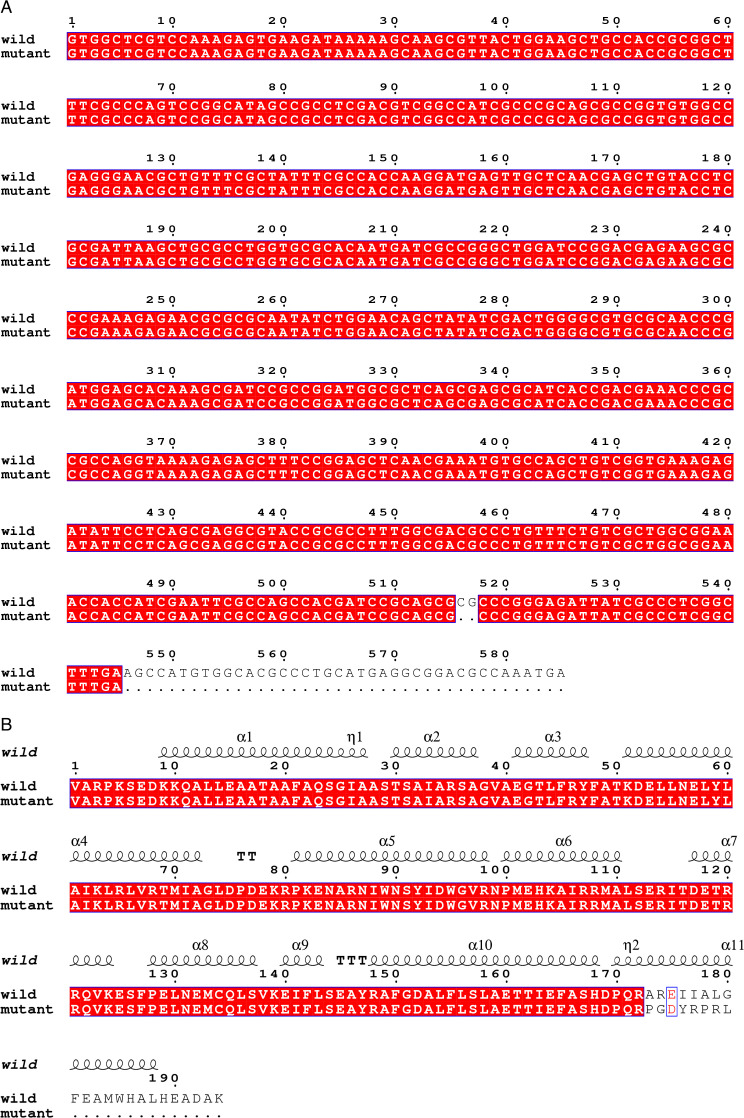
Molecular characterization of the *ramR* mutation. (**A**) DNA sequence alignment comparing the wild-type strain and the mutant strain, highlighting the specific mutation site. (**B**) Protein sequence alignment demonstrating the impact of the mutation on the RamR amino acid sequence, including the frame-shift mutation and premature termination.

Compared to the parental strain ATCC 43816, we identified three mutant strains, cultured in broth with relatively high concentrations of tigecycline (4, 8, and 16 mg/L), which all carried the same mutation (g. 517_518 del) in *ramR*. The sequence containing the mutant sites was initially determined by PCR and Sanger sequencing (Fig. 1A). The translation of the amino acid sequence exhibited a misalignment starting at position 173, resulting in premature termination of the protein (Fig. 1B) (20).

### Antimicrobial susceptibility testing of *ramR* deletion strain and complementary strains

Complementation of the wild-type *ramR* gene into ATCC 43816Δ*ramR* restored bacterial sensitivity to tigecycline. The frame-shift mutation in *ramR* resulted in an eightfold increase in the MIC of *Klebsiella pneumoniae* to tigecycline. This finding suggests that the truncation of the final helix in the RamR protein is associated with enhanced resistance (Table 5). In conjunction with the previous results (Table 4), three induced strains cultured in broth with relatively low concentrations of tigecycline (0.5, 1, and 2 mg/L) did not harbor changes in RamR amino acid residues, yet their MIC values still increased compared to pre-induction levels. The Δ*ramR* strain displayed a significant increase in tigecycline resistance; however, the induced strain ultimately had a higher tigecycline MIC than the knockout strain. Thus, the non-synonymous mutation of the *ramR* gene cannot be regarded as the sole factor contributing to tigecycline resistance in *Klebsiella pneumoniae*.

**TABLE 5:** MICs of *ramR* deletion strain and complementary strains to tigecycline

Isolate*^a^*	MIC (mg/L) (susceptibility)
ATCC 43816	1 (S)
ATCC 43816-pBAD24	1 (S)
ATCC 43816Δ*ramR*	8 (R)
ATCC 43816Δ*ramR-*pBAD24	8 (R)
ATCC 43816Δ*ramR*-pC*ramR*	1 (S)
ATCC 43816Δ*ramR*-pCt*ramR*	8 (R)

^*a*^ Δ, deletion. t*ramR* represents mutated *ramR* sequence.

### High expression of *ramR* after ATCC 43816 was induced by tigecycline

**Fig 2:**
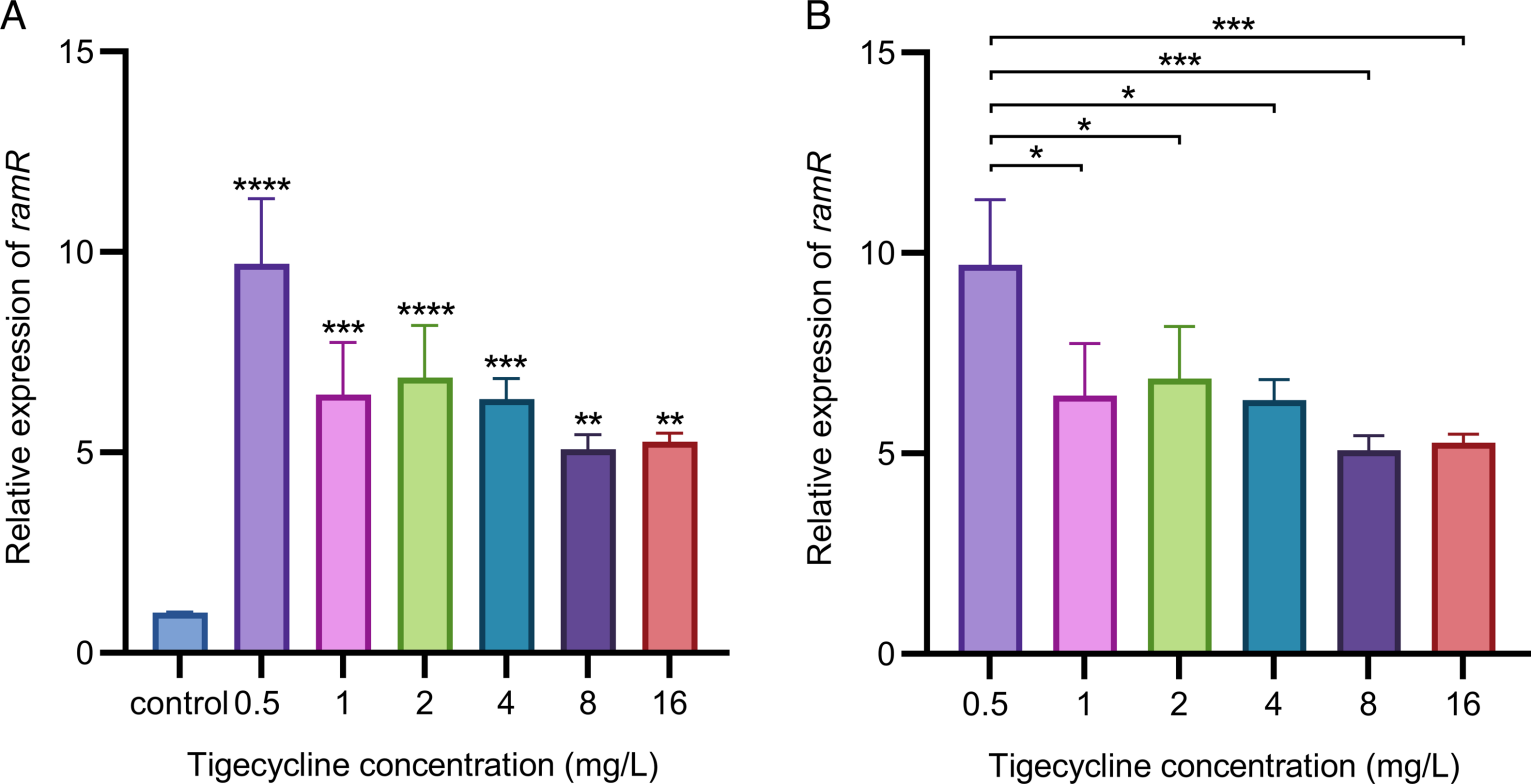
Expression analysis of *ramR* in tigecycline-induced strains. Relative expression levels of *ramR* in the wild-type strain ATCC 43816 and strains induced with varying concentrations of tigecycline (0.5, 1, 2, 4, 8, and 16 mg/L). The delta CT values and SDs are shown. (*, *P* < 0.05; **, *P* < 0.01; ***, *P* < 0.001; ****, *P* < 0.0001. Ordinary one-way ANOVA. CT, cycle threshold, SD, standard deviation.) (**A**) * indicates significant differences between each induced strain and the uninduced control. (**B**) * above the connecting lines indicate significant differences between the 0.5 mg/L tigecycline-induced strain and other induced strains.

After induction, the expression levels of *ramR* in all strains were significantly higher than before (*P* < 0.01) (Fig. 2A). The expression level of *ramR* peaked at a tigecycline concentration of 0.5 mg/L, then decreased, showing differences compared to the other induction concentrations (*P* < 0.05). However, no statistically significant differences in the *ramR* expression levels were observed across tigecycline concentrations ranging from 1 to 16 mg/L (comparisons were made between the mean values of each concentration group) (Fig. 2B).

### Resistance stability testing

**Fig 3:**
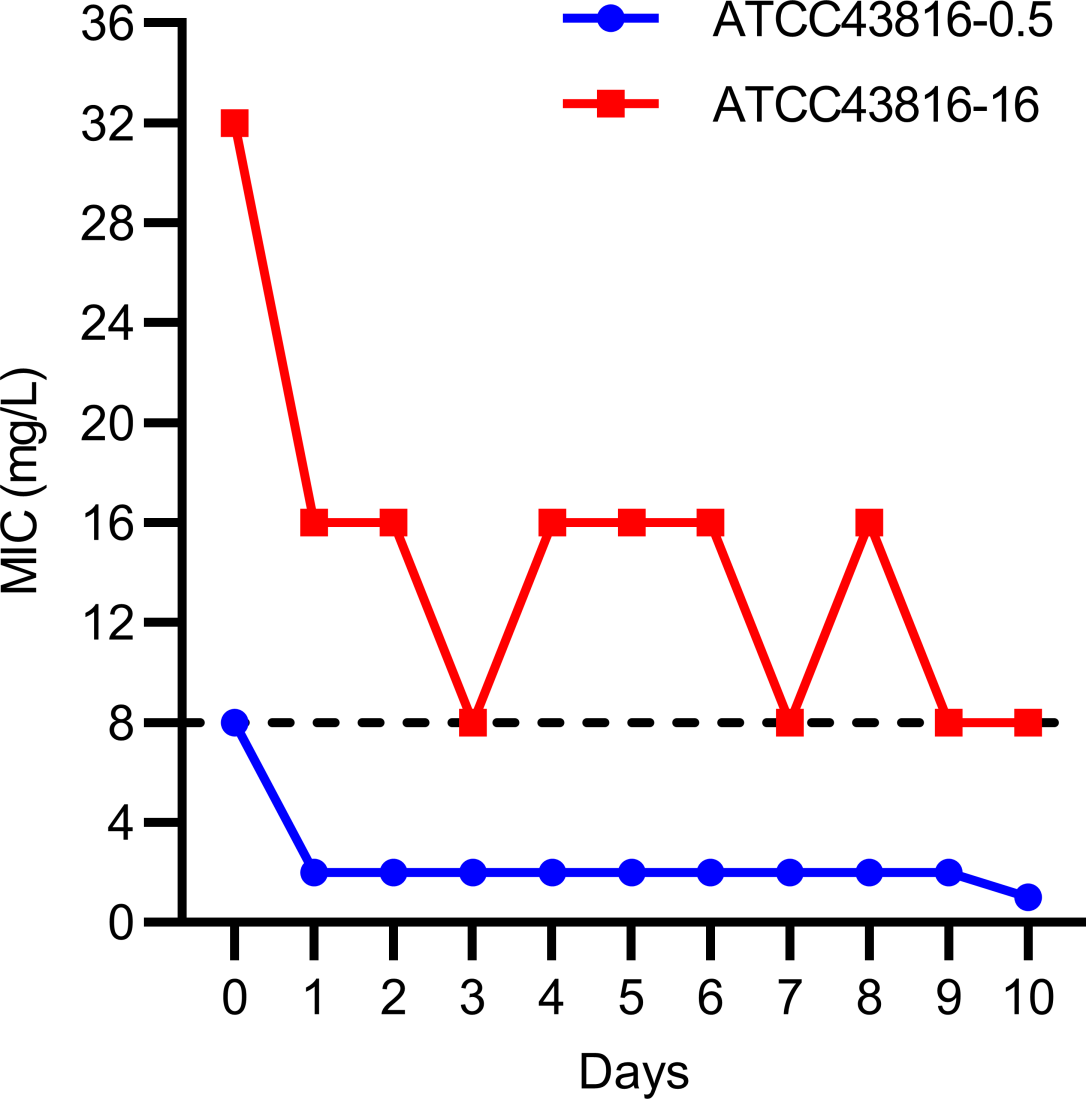
Stability of tigecycline resistance in induced strains. Long-term stability of tigecycline resistance was assessed by serially culturing ATCC 43816-0.5 and ATCC 43816-16 strains for 200 generations. MIC values were measured at regular intervals to monitor resistance stability over time.

To evaluate the resistance stability of ATCC 43816-0.5 and ATCC 43816-16, both strains were continuously cultured without tigecycline. As shown in Figure 3, ATCC 43816-0.5 became sensitive to tigecycline (MIC = 1 mg/L) after 200 generations of cultivation, while ATCC 43816-16 remained resistant to tigecycline (MIC = 8 mg/L) with the same mutation in *ramR* detected. The loss of resistance in ATCC 43816-0.5 (the *ramR* unmutated strain) indicates that sustained tigecycline pressure is necessary to maintain high MIC values. Conversely, ATCC 43816-16 (the *ramR* mutant strain) retained its resistance (MIC = 8 mg/L), suggesting that the drug resistance phenotype is stable and the mutation persists within the strain.

### Homology modeling and mutation site labeling

**Fig 4:**
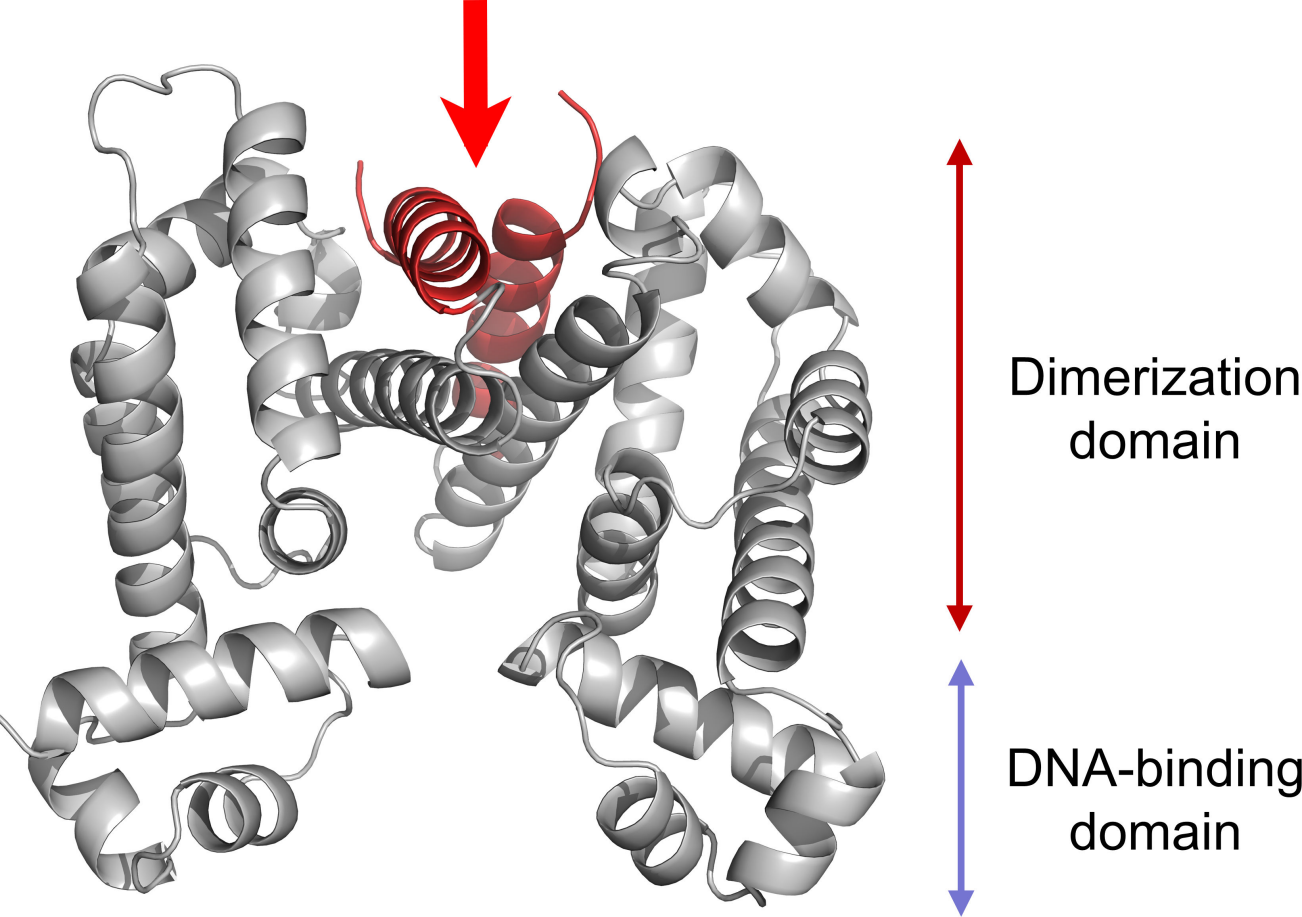
Homology modeling of the RamR protein structure. The RamR protein structure, generated by homology modeling, is shown with the mutation site highlighted (red arrow).

The RamR amino acid sequence of ATCC 43816 was submitted to the SWISS-MODEL workspace for homology modeling, using the structure of RamR from *Salmonella Typhimurium* (PDB ID: 3vvy) as a template. The sequence similarity of modeling alignment was 48%, meeting the criteria for reliable structure prediction (21), which enabled us to obtain a simulated tertiary structure. The resulting structure was visualized with PyMOL 3D graphics software, highlighting the mutation site in red. As shown in Figure 4, the base deletion in mutant strains was located in the dimerization domain.

## DISCUSSION

It has been reported that RamR can inhibit the expression of AcrAB-TolC efflux pump genes after binding to the *ramA* promoter (9) by suppressing the transcription of *ramA*. Other studies have identified that mutations at specific sites in *ramR*, such as Q122*, Δ190 bp (322–511), A40T, L58P, Q135*, and S29* (*, stop codon; Δ, deletion), can weaken the inhibitory effect on *ramA*, leading to its overexpression and resulting in resistance to antibiotics like tigecycline (22).

This study reported, for the first time, a novel *ramR* mutation that contributes to tigecycline resistance, mediated through impaired function of the RamR protein. Under progressively increasing tigecycline pressure, ATCC 43816 initially managed tigecycline efflux by upregulating *ramR* expression. However, with a further increase in tigecycline concentration, a g. 517_518 del mutation occurred in *ramR*, leading to the loss of inhibition of tigecycline efflux and a corresponding decrease in its expression, ultimately establishing a stable tigecycline resistance phenotype.

RamR is composed of two monomers, each containing two distinct regions, the DNA-binding domain and the dimerization domain. The first three alpha-helices of the RamR secondary structure comprise the DNA-binding domain, while the last six alpha-helices form the dimerization domain (23). In this study, sequence alignment and three-dimensional (3D) modeling of the RamR protein indicate that the base deletion of the mutant strain occurs within the functional domain of RamR dimerization, potentially impairing the dimerization of RamR and decreasing the DNA-binding ability.

This study also found that many strains could not maintain stable tigecycline resistance in the absence of the antibiotic. Among them, *ramR* g. 517_518 del mutant strains formed under high tigecycline concentrations will remain resistant, as the mutation can stably persist in the strain.

This study unveils a novel mechanism driving tigecycline resistance, offering critical insights into its molecular underpinnings. We identified a unique frame-shift mutation (g. 517_518 del) in the *ramR* gene of *Klebsiella pneumoniae*, which results in structural truncation of the RamR protein and significantly impairs its dimerization and DNA-binding capabilities. This molecular alteration disrupts the protein’s regulatory function, leading to the overexpression of efflux pump components and the subsequent development of a stable tigecycline resistance phenotype across multiple generations. The persistence of this resistance phenotype, even in the absence of antibiotic pressure, underscores its potential clinical significance in the context of antibiotic resistance evolution.

These results not only advance our fundamental understanding of resistance mechanisms but also offer valuable translational implications. The identified molecular signature serves as a potential target for the development of novel therapeutic interventions, including small molecule inhibitors and peptide-based therapies designed to restore RamR function or counteract the overactive efflux pumps. Furthermore, this work contributes to the rational design of next-generation antimicrobial agents through structure-based drug discovery approaches, aligning with contemporary strategies in antibiotic development.

In future investigations, we aim to broaden our analysis by incorporating additional strains into the induction process, with a particular focus on clinically relevant isolates. Through extensive screening of these clinical strains, we seek to elucidate the prevalence and distribution of the identified mutations. These findings will not only inform the design of TaqMan probes for rapid detection but also facilitate the development of targeted therapeutic strategies to combat tigecycline resistance.

In conclusion, this study elucidates a novel resistance mechanism mediated by *ramR* mutation and highlights its potential clinical relevance. By bridging fundamental research with translational applications, our work contributes to the ongoing global effort to address the growing challenge of antibiotic resistance, offering new avenues for therapeutic and diagnostic innovation.

## Data Availability

The Whole Genome Shotgun project has been deposited at DDBJ/ENA/GenBank under the accession JAVKOT000000000. The version described in this paper is version JAVKOT010000000. The GenBank accession number of the *ramR* mutant sequence is OR509661.
